# Effect of High Intensity Interval Training on Matrix Metalloproteinases in Women with Breast Cancer Receiving Anthracycline-Based Chemotherapy

**DOI:** 10.1038/s41598-020-61927-x

**Published:** 2020-04-03

**Authors:** Kyuwan Lee, Irene Kang, Wendy J. Mack, Joanne Mortimer, Fred Sattler, George Salem, Christina M. Dieli-Conwright

**Affiliations:** 1 Division of Cardiology, Children’s Hospital Los Angeles, Keck School of Medicine, University of Southern California (USC), Los Angeles, CA 90089 USA; 2 Department of Medicine, Keck School of Medicine, USC, Los Angeles, CA 90089 USA; 3 Department of Preventive Medicine, Keck School of Medicine, USC, Los Angeles, CA 90089 USA; 4 Division of Medical Oncology & Experimental Therapeutics, City of Hope Comprehensive Cancer Center, Duarte, CA 91010 USA; 5 Division of Biokinesiology and Physical Therapy, Ostrow School of Dentistry, USC, Los Angeles, CA 90089 USA; 6 Division of Population Sciences, Dana-Farber Cancer Institute, Harvard Medical School, Boston, MA 02215 USA

**Keywords:** Physiology, Cardiology, Health care, Oncology, Risk factors

## Abstract

Anthracycline chemotherapy is commonly used to treat breast cancer yet may increase the level of matrix metalloproteinases (MMP) -2 and -9, which increase the risk of atherosclerosis. While exercise has been shown to reduce the level of MMP in patients with diabetes, high intensity interval training (HIIT) has not been utilized to improve level of MMP in women with breast cancer receiving anthracycline chemotherapy. Thirty women were randomized to either 8-week HIIT or control (CON) group. The CON group was offered the HIIT intervention after 8 weeks. MMP-1, -2 -7, -9, tissue inhibitor of MMP (TIMP) -1, and-2 were measured at baseline and post-intervention. Repeated measures ANCOVA and paired t-test were performed to assess changes in MMP and TIMP. Post-intervention, no significant between-group differences were observed for MMP and TIMP. However, within-group decrease in MMP-9 was observed in the HIIT group [104.3(51.9) to 65.2(69.1); P = 0.01]. MMP-9 in the CON group was not significantly changed [115.5(47.2) to 90.4(67.9);]. MMP-2 significantly increased in both the HIIT group [76.6(11.2) to 83.2(13.1); P = 0.007) and the CON group [69.0(8.9) to 77.6(11.1) P = 0.003). It is unclear whether an 8-week HIIT intervention influences MMP-9 in breast cancer patients undergoing anthracycline chemotherapy. Additional investigations are required to understand the exercise-induced changes in MMP-2 and -9 in women undergoing anthracycline chemotherapy.

## Introduction

Anthracycline-based chemotherapy is one of the most commonly used neoadjuvant or adjuvant chemotherapies for the treatment of breast cancer, yet its clinical use has been associated with cardiovascular toxicity, most notably as a decrease in left ventricular ejection fraction more than 10% or to cutoff value of 53%^1,2^. The cardiovascular toxicity of anthracycline-based chemotherapy is associated with oxidative stress which degrades the extracellular matrix (ECM) structure within the vascular system^3^. The ECM surrounding vascular endothelial cells, also known as endothelial ECM, is primarily regulated by the enzymes matrix metalloproteinases (MMP) and tissue inhibitor of MMPs (TIMP)^4,5^. The overexpression of certain MMPs (i.e., MMP-2 and -9) is correlated with increased coronary plaque (r = 0.49, P = 0.007) in patients with coronary artery disease^6^. MMP-2 has been reported higher in patients with metabolic syndrome compared to healthy controls (3,452 ± 429 vs 4,263 ± 427)^7^. MMP-9 was significantly associated with high blood pressure [beta: 2.9 (0.4–5.4), low HDL [-2.5 (-5.0–0.0), triglycerides (best 3.7 (1.4–6.1) and overall cardiovascular risk [4.7 (2.6–6.8) after adjusting for age, and sex^8^. Further, increased levels of MMP-9 promote plaque development in the vasculature^9^, and elevated levels of MMP-9 has been found in patients with hypertension than normotensive controls (2.31 ± 0.2 vs 1.94 ± 0.3)^10^ and correlated with higher femoral atherosclerosis (r = 0.16; P < 0.01)^11^. Moreover, anthracycline-based chemotherapy increases oxidative stress, measured by the level of reactive oxygen species, which causes the overexpression MMP-2 and MMP-9^12–14^. The adverse nature of anthracycline-induced alterations in ECM-regulating enzymes provides a strong rationale for the design and testing of intervention strategies (e.g. exercise) to improve vascular endothelial function, and ultimately reduce chemotherapy-related cardiovascular mortality in breast cancer patients^15,16^.

MMP-2 and -9 are reduced with exercise interventions in patients with type 2 diabetes^17^ and breast cancer^18^; other MMPs such as MMP-1, -7, -9, TIMP-1 and -2, have not been determined whether exercise improves the level of MMPs in clinical populations such as type 2 diabetes. While there is evidence showing that moderate-intensity aerobic exercise training may decrease MMP-2 and -9 levels in clinical research settings^17,18^, the effects of high intensity interval training (HIIT) on MMP levels have not been investigated in any population. HIIT is an exercise strategy that maximizes exercise intensity by using bursts of concentrated effort alternated with recovery periods^19^. HIIT allows patients to perform vigorous intensity exercise due to the ‘on-off’ pattern of the activity. The “on” portion of HIIT typically involves 1–4 minutes performed at 80–90% of peak power output (PPO), followed by the “off” period (2–3 minutes of active break [10% PPO])^20^. This interval-based strategy has been shown to be more effective than moderate continuous intensity aerobic exercise for improving endothelial function among individuals with stroke or coronary artery disease^21–23^, and reduces liver fat and increases early diastolic filling rate in patients with non-alcoholic fatty liver disease^24^.

The aim of this pilot study was to determine the effects of an 8-week HIIT intervention on ECM-regulating enzymes in early stage breast cancer patients receiving anthracycline-based chemotherapy. We also explored the comparative changes in other MMP and TIMP, including MMP-1, -7, -10, and TIMP-1 and -2. We hypothesized that a group of participants that performed the 8-week HIIT intervention would demonstrate greater decreases in circulating serum MMP-2 and -9 compared to a group of participants that did not perform HIIT (CON).

## Methods

Detailed methods have been published^25^. In brief, this pilot study compared HIIT group versus CON group on baseline to post-intervention changes in ECM-regulating enzymes. Participants were recruited from the breast cancer clinics at the University of Southern California (USC) Norris Comprehensive Cancer Center (NCCC) and the Los Angeles County Medical Center. Following written informed consent, eligible participants completed baseline tests (week 0) within one week prior to the start of the intervention. Participants were randomly assigned by computer-generated, investigator-blinded randomization assignments; randomization was stratified by neoadjuvant versus adjuvant anthracyclines treatment in a 1:1 allocation ratio by the Clinical Investigation Support Office at the NCCC. Participants randomized to the HIIT group completed the exercise sessions for a total of 90 min of weekly exercise during the 8-week intervention period. Participants randomized to the CON group were asked to maintain their current level of physical activity, which was defined as less than 30 min of total exercise per week. All participants returned within 1 week following completion of the 8-week study period for post-intervention (week 9) testing. For ethical reasons related to the overall beneficial effect of exercise and to encourage participation, participants in the CON group were offered the same HIIT intervention following the 8-week study period.

### Study population

Participants were recruited if women were over 18 years of age diagnosed stage I-III breast cancer; planned to initiate neoadjuvant or adjuvant anthracycline chemotherapy within 1 week of the start of anthracycline-based chemotherapy. In addition, participants were currently participating in less than 30 minutes of physical activity per week, were not smoking during previous 12 months, and willing to travel to the exercise facility at USC. Physician clearance was provided to participate in the exercise program. We excluded participants if women had a history of chronic disease including diabetes, uncontrolled hypertension or thyroid disease; weight reduction >10% within past 6 months; metastatic disease; overt cardiovascular diseases (myocardial infarction, stroke, angina). Further contra-indications to exercise or participation in regular exercise were confirmed to exclude potential participants. Recruitment period was between August 15^th^, 2017 and August 6^th^, 2018. The protocol and informed consent were IRB-approved (HS-15-00227) and registered (ClinicalTrials.gov: NCT02454777; date of registration: May 27^th^, 2015). All methods were carried out in accordance with relevant guidelines and regulations along with the approval and informed consent.

### Exercise intervention

Participants assigned to the HIIT group were asked to perform supervised one-on-one exercise sessions 3 times/week using a stationary bicycle (Life Fitness 95 Elevation Series, Rosemont, IL). Prior to initiating the exercise intervention, relative exercise intensity was individually prescribed for each HIIT participant based on peak power output (PPO; watts) performed at 60 rpm, which was measured by a VO_2_max fitness test performed on a stationary bike^23^. PPO was defined as the highest power output generated during a maximal cycling test^22^. Each exercise session consisted of a 5-minute warm-up performed at 10% PPO, followed by a 20-minute HIIT protocol. Based on previous studies that reported significant improvements of cardiovascular function with high compliance to the HIIT intervention^22,23^, the HIIT protocol consisted of 7 bouts of 1 minute high-intensity exercise (90% of PPO) followed by 2 minutes of low intensity (10% of PPO). Participants were asked to rest at least 24 hours before the next session and to complete their sessions on days when they did not receive chemotherapy infusions. We documented power output, heart rate, rating of perceived exertion (RPE; rated on the Borg scale of 6–20), and total minutes of exercise for each session. Participants were encouraged to make up any missed sessions within the same week. The CON group was asked to maintain their level of physical activity during the 8-week study period. Following the 8-weeks of intervention, we offered the same HIIT intervention to the CON group.

### Outcome measures

#### MMP and TIMP

A fasting venous blood sample was obtained from each participant by a licensed phlebotomist. Blood samples were centrifuged at 3,000 rpm for 10 minutes, and the serum portion was pipetted into 0.5 mL aliquots using a sterile pipette. Aliquots were stored at −80 °C until further processing. Participants in the HIIT group were requested to avoid any strenuous physical activities for at least 48 h before blood sampling at baseline (week 0) and post-intervention (week 9). MMP-1, -2, -7, -9, and -10 concentrations were examined as a single batch at study completion and processed using the magplex suspension bead array immuno-assays on a Luminex 100 Bioanalyzer (Luminex 100, Luminex Corporation, Austin, Texas) according to the kit manufacturer’s instructions (Milliplex ELISA kits, Millipore, MA) by the Metabolism Core Laboratory/Human Physiology laboratory of the University of Alabama Birmingham. Specifically, 50 ml of each serum sample were incubated with fluorokine-coloured microspheres coated with specific antibodies, and analytes were allowed to bind to the specific antibody-coated microspheres. Samples were then washed and incubated with biotinylated antibodies and phycoerythrin-conjugated streptavidin. Finally, fluorescence was detected using a flow cytometry technique (Luminex 100, Luminex Corporation, Austin, Texas). All samples were assayed in triplicate wells (25 *u*L per well), and the mean of these results was used for statistical analysis. MMP concentration was calculated by reference to an eight-point spline fit curve.

#### Statistical analyses

Baseline participant characteristics were summarized by descriptive statistics. Distribution of outcomes were evaluated and presented as mean (SD) for continuous outcomes and frequency (%) for categorical outcomes. Normality was evaluated by the Kolmogorov-Smirnov test. If data were not normally distributed, non-parametric corollary for continuous outcomes and χ2 test for categorical outcomes were used. As there was no influence of outliers, data points were not removed from the original dataset. Group comparisons of baseline participant characteristics were made using t-test or non-parametric corollary for continuous outcomes and χ^2^ test for categorical outcomes^26^. Participant baseline characteristics that were different across groups were included as covariates in the statistical analyses. Given the small sample size (N = 30), baseline variables with a difference of P < 0.10 were considered as additional covariates after testing for collinearity. An analysis of covariance (ANCOVA) model adjusting for the baseline value of the outcome was performed with treatment group (HIIT vs CON) and time (baseline/post-intervention) as factors^27^. For within group difference, the changes in MMP from baseline to week 9 were examined by a paired t-test, with a level of significance set at P < 0.05^28^. Repeated measures ANCOVA on the trial outcomes was a 2 (group: HIIT, CON) x 2 (time: baseline, post-intervention) analysis. Cohen’s d effect size (d) for the changes in MMP was calculated using the difference in means from baseline and post-intervention and the pooled standard deviations^29^. All analyses were performed with SPSS (v.22).

### Ethics approval and consent to participate

The protocol and informed consent were approved by the University of Southern California Institutional Review Board (HS-1500227).

## Results

Fifty-eight women were screened for eligibility and 30 participants were enrolled, consented, and randomized to the HIIT (n = 15) or CON groups (n = 15). Baseline characteristics and CONSORT diagram have been published previously^30^. Briefly, participants were mainly Hispanic white (73%) and BMI was 31.0 ± 7.5 kg/m^2^. The majority of participants were diagnosed with stage II (30%) or III (63%) breast cancer and received neoadjuvant chemotherapy (77%). High attendance to the 8-week HIIT intervention was 82.3% attained by the HIIT group^31^. The average range of RPE during HIIT was between 15–17 on the 6–20 scale among the participants. There were no adverse events reported over the duration of the intervention.

ECM-regulating enzyme results are displayed in Table 1. At baseline there were no differences in MMP (-1, -2, -7, -9, -10), TIMP-1 or TIMP-2 between the two groups. There was a significant within-group decrease in MMP-9 in the HIIT group [104.3 (51.9) to 65.2 (69.1); −37.4%; P = 0.01; d = 0.20) while MMP-9 in the CON group was not significantly changed [115.5 (47.2) to 90.4 (67.9); −21.7%; P = 0.10). Unexpectedly, a significant within-group increase in MMP-2 was observed in both HIIT group (8.6%; P = 0.007) and the CON group (12.6%; P = 0.003). Post-intervention, there was no significant within group or between group change in MMP-1, -7, and -10.

**Table 1:** Comparison of MMPs and TIMPs between HIIT and CON groups.

Variable	Baseline, mean (SD)	Post-intervention		Between Group Difference Post intervention	
	Mean (SD)	Mean (SD)	*P*	Mean (95% CI)	*P*
**MMP-1 (ng/ml)**				4.5 (−2.1 to 11.1)	0.98
HIIT	14.9 (11.7)	19.4 (12.4)	0.17		
CON	14.3 (19.2)	18.8 (21.6)	0.14		
**MMP-2 (ng/ml)**				6.5 (2.1 to 10.9)	0.53
HIIT	76.6 (11.2)	83.2 (13.1)	0.007*		
CON	69.0 (8.9)	77.6 (11.1)	0.003*		
**MMP-7 (ng/ml)**				−0.05 (−0.64 to 0.54)	0.33
HIIT	4.4 (0.9)	4.3 (0.8)	0.85		
CON	4.8 (2.6)	4.3 (2.7)	0.13		
**MMP-9 (ng/ml)**				−46.4 (−80.4 to 12.4)	0.65
HIIT	104.3 (51.9)	65.2 (69.1)	0.01*		
CON	115.5 (47.2)	90.4 (67.9)	0.10		
**MMP-10 (ng/ml)**				0.02 (−0.4 to 0.5)	0.61
HIIT	1.4 (0.7)	1.4 (0.5)	0.91		
CON	1.7 (1.4)	1.5 (0.9)	0.62		
**TIMP-1 (ng/ml)**				15.3 (−54.4 to 84.9)	0.67
HIIT	362.2 (92.5)	377.5 (91.5)	0.65		
CON	322.3 (92.6)	354.3 (87.3)	0.17		
**TIMP-2 (ng/ml)**				0.4 (−7.2 to 7.9)	0.46
HIIT	93.3 (12.6)	93.7 (12.1)	0.92		
CON	86.3 (10.9)	89.8 (14.5)	0.11		

HIIT, high intensity interval training; CON, control; MMP, matrix metalloproteinases, TIMP, tissue inhibitor of matrix metalloproteinases.

*P value denotes significant change in outcome measure at the p < 0.05 level by paired t-test comparing changes in the HIIT group from baseline to post-intervention, and in the CON group from baseline to post-intervention.

## Discussion

Our results examined for the first time whether an 8-week HIIT exercise intervention influences ECM-regulating enzymes in cancer patients. While there was no significant between-group difference, an 8-week HIIT intervention significantly (within group differences) reduced MMP-9 levels in breast cancer patients undergoing anthracycline-based chemotherapy yet increased MMP-2 levels; no significant changes in MMP-1, -7, -10, TIMP-1 and TIMP-2 were observed. To our knowledge, this is the first study to examine exercise-related change in MMP-9 in cancer patients undergoing anthracycline-based chemotherapy.

The change in MMP-9 reported here is similar to previous exercise studies in non-cancer^17^ and cancer populations^18^ (the latter was an observational study). Kadoglou *et al*. (2010) reported a significant within-group decrease in serum MMP-9 after a 16-week supervised endurance training program (30–60 min of brisk walking; 4 days/week) in patients with type 2 diabetes^17^. In the observational study, Giganti *et al*. (2016) found that breast cancer patients who completed cancer treatment (10 years post-surgery) and who regularly participated in exercise (30 min treadmill plus 20 min strength training; 3 times a week) had significantly lower MMP-9 levels compared to breast cancer patients who did not participate in regular exercise^18^. However, this study lacked a baseline measure of MMP levels and did not involve a direct exercise intervention. Although it is difficult to compare our findings with the previous studies due to different exercise protocols, difference assessment of biomarkers and disease status (i.e. breast cancer vs type 2 diabetes), our study suggests that HIIT may partly contribute to the level of MMP-9 with a lower volume of exercise (90 min vs 150 min per week) over fewer number of weeks (8 vs 16 weeks). Further, we did not measure proBNP and cardiac troponinT; these serum biomarkers have been shown to predict cardiotoxicity in cancer patients^32^. It is noted that future clinical exercise trials may elucidate the effects of exercise on these cardiotoxicity markers in conjunction with the expression of MMP levels.

Potential biologic mechanisms may explain the exercise-induced changes in MMP-9. While we did not measure the level of inflammatory markers, a previous study reported that exercise training did not counteract the increase in inflammation, suggesting that beneficial effects of exercise on cardiotoxicity during breast cancer chemotherapy may not be regulated by inflammatory markers^33^. It is plausible that exercise may have reduced oxidative stress, thereby reducing MMP-9 levels^34^. Previous studies reported that MMP-9 was reduced in conjunction with tumor necrosis factor-α (TNF-α) and interleukin-6 (IL-6) with no change in MMP-2^35,36^. Evidence derived from a rat model indicated that MMP-9 expression is elevated by oxidative stress^34,37,38^, suggesting that the oxidative stress molecules^38,39^ may increase MMP-9 synthesis. It is possible that oxidative stress in the ECM were reduced by HIIT, which subsequently reduced MMP-9 levels. Future investigations are warranted to assess exercise-induced changes in oxidative stress with ECM-regulating enzymes to understand mechanism by which exercise influences MMP and TIMP levels.

While we hypothesized that HIIT would reduce MMP-2 levels in breast cancer patients undergoing anthracycline-based chemotherapy, MMP-2 levels were significantly increased in both the HIIT and CON groups. Although increases in MMP-2 levels are expected in patients undergoing anthracycline-based chemotherapy^40,41^, we expected the HIIT intervention would offset this increase. Thus, it appears that the exercise intervention was not adequate to offset the negative influence of anthracycline-based chemotherapy on this enzyme. This finding is consistent with a previous finding of Kadoglou *et al*.^17^ in that a 16-week supervised endurance training did not alter MMP-2 level in type 2 diabetics. In addition, Donley *et al*. (2014) reported that an 8-week aerobic exercise training (3 times/week, 60 min per day, 60–85% heart rate reserve) in cohorts with metabolic syndrome also did not change MMP-2 level^7^. Therefore, exercise, in general, may not be an effective non-pharmacologic strategy to target MMP-2 in breast cancer patients undergoing anthracycline-based chemotherapy. It is difficult, however, to compare these studies due to the variability across exercise protocols and clinical cohorts involved. Factors which still need to be considered before concluding that MMP-2 does not respond to exercise include the type, duration, frequency, and intensity of the exercise program, as well as the age and health status, of the cohort. Future studies are needed to examine how these multiple factors will influence MMP-2 levels in breast cancer patients who completed anthracycline-based chemotherapy.

Previous studies suggest that MMP-1, -7, -10 have a negative impact on cardiovascular disease such as hypertension^42^ or type 2 diabetes^42–44^. However, the present study did not find any significant changes in MMP-1, -7, -10 indicating that MMP-1, -7, -10 are not responsive to anthracycline-based chemotherapy and/or exercise training. A previous exercise intervention study in cohorts with metabolic syndrome conducted by Donley *et al*. reported findings consistent with our study showing that MMP-1, -7, -10 were not changed following an 8-week of aerobic exercise training^7^. Additional investigations are needed to elucidate the underlying exercise-induced effects on MMP-1, -7, -10.

Levels of TIMP-1 and -2 also did not change in either group implying that TIMP may not be influenced by anthracycline-based chemotherapy and/or HIIT. Our data suggest that the activity of TIMP is not influenced by anthracycline-based chemotherapy and/or HIIT. Our results are consistent with Donley *et al*. (2014) which determined that TIMP-1 and -2 were not significantly changed with exercise training in patients with metabolic syndrome^7^. Future studies are necessary to confirm whether TIMP-1 and -2 levels can be influenced by exercise and/or anthracycline-based chemotherapy.

The strengths of our study included: (1) direct one-on-one supervision of all exercise sessions, (2) high adherence of 82.3%; previous studies investigating exercised-related changes in MMP did not report the adherence to exercise^7,17,18^; (3) focus on a single chemotherapeutic agent so the effects of exercise on MMP with chemotherapy could be better understood.

Despite these strengths, we acknowledge the following limitations; (1) this study was designed as a pilot study, thus the small sample size is notable; (2) other related biomarkers were not investigated; (3) our patient population was mostly obese and may have been insulin resistant therefore, it is possible that our findings may be the indirect result of improvements in insulin sensitivity caused by exercise^45^.

In conclusion, it is unclear whether HIIT influences levels of MMP in breast cancer patients undergoing anthracycline-based chemotherapy yet MMP-2 was significantly increased both the HIIT and CON group; with no changes on MMP-1, -7, -10, TIMP-1 and -2. Further studies with an adequately powered sample size are required to identify an effective approach preventing side effects on ECM-regulating enzymes in breast cancer patients undergoing anthracycline–based chemotherapy.

## Data Availability

The datasets used and/or analyzed during the current study are available from the corresponding author on reasonable request.
